# Screen time and manic symptoms in early adolescents: prospective findings from the Adolescent Brain Cognitive Development Study

**DOI:** 10.1007/s00127-025-02814-6

**Published:** 2025-02-19

**Authors:** Jason M. Nagata, Gabriel Zamora, Abubakr A.A. Al-Shoaibi, Jason M. Lavender, Kyle T. Ganson, Alexander Testa, Jinbo He, Fiona C. Baker

**Affiliations:** 1 Department of Pediatrics, University of California, San Francisco, San Francisco, CA USA; 2 Military Cardiovascular Outcomes Research Program (MiCOR), Department of Medicine, Uniformed Services University of the Health Sciences, Bethesda, MD USA; 3 The Metis Foundation, San Antonio, TX USA; 4 Factor-Inwentash Faculty of Social Work, University of Toronto, Toronto, ON Canada; 5 Department of Management, Policy and Community Health, University of Texas Health Science Center at Houston, Houston, TX USA; 6 Division of Applied Psychology, School of Humanities and Social Science, The Chinese University of Hong Kong, Shenzhen, Guangdong China; 7 Center for Health Sciences, SRI International, Menlo Park, CA USA; 8 School of Physiology, University of the Witwatersrand, Johannesburg, South Africa

**Keywords:** Screen time, Adolescents, Media, Mania, ABCD

## Abstract

**Purpose:**

This study aimed to examine prospective associations between screen time and manic symptoms in early adolescents, and the extent to which problematic screen use (characterized by addiction, conflict, relapse, and withdrawal) mediates the association.

**Methods:**

We analyzed prospective cohort data from the Adolescent Brain Cognitive Development Study (*N* = 9,243; ages 10–11 years in Year 1 in 2017–2019; 48.8% female; 44.0% racial/ethnic minority). Participants reported daily time spent on six different screen subtypes. Linear regression analyses were used to determine associations between typical daily screen time (Year 1; total and subtypes) and manic symptoms (Year 3, 7 Up Mania scale), adjusting for potential confounders. Sleep duration, problematic social media use, and problematic video game use (Year 2) were tested as potential mediators.

**Results:**

Adjusting for covariates, overall typical daily screen time in Year 1 was prospectively associated with higher manic symptoms in Year 3 (B = 0.05, 95% CI 0.03, 0.07, *p* < 0.001), as were four subtypes: social media (B = 0.20, 95% CI 0.09, 0.32, *p* = 0.001), texting (B = 0.18, 95%CI 0.08, 0.28, *p* < 0.001), videos (B = 0.14, 95% CI 0.08, 0.19, *p* < 0.001), and video games (B = 0.09, 95% CI 0.04, 0.14, *p* = 0.001). Problematic social media use, video game use, and sleep duration in Year 2 were found to be significant partial mediators (47.7%, 58.0%, and 9.0% mediation, respectively).

**Conclusion:**

Results indicate significant prospective relationships between screen time and manic symptoms in early adolescence and highlight problematic screen use, video game use, and sleep duration as potential mediators. Problematic screen use may be a target for mental health prevention and early intervention efforts among adolescents.

**Supplementary Information:**

The online version contains supplementary material available at 10.1007/s00127-025-02814-6.

## Introduction

Excessive screen time has become commonplace among American youth, school-aged children, and adolescents alike [1]. In the United States, the average adolescent spends over 8 h on screens daily, representing a doubling of pre-pandemic estimates [2, 3]. There has also been an increase in mental health concerns and treatment demand among American youth, with 29% experiencing mental health problems based on meta-analyses from 2019 to 2020 [4]. Notably, evidence supports an association between screen time and deleterious psychological outcomes, including depression and anxiety [5, 6].

While the association between screen time and affective disturbances, in particular, has been well studied, the relationship between screen time and manic symptoms remains unexplored, especially among early adolescents [7]. Manic symptoms include inflated self-esteem or grandiosity, decreased need for sleep, pressured speech, flight of ideas, distractibility, increased goal-directed activity, and excessive involvement in pleasurable activities [8]. Episodes of mania and hypomania, which are core diagnostic criteria for bipolar-spectrum disorders, are characterized by distinct periods of abnormally and persistently elevated, expansive, or irritable mood and abnormally and persistently goal-directed behavior or energy [9].

Adolescence represents a vulnerable developmental period concerning the onset of bipolar-spectrum disorders, and earlier onset is associated with worse functional outcomes and increased symptomatic morbidity [10, 11]. Adolescents with bipolar disorder report a lower quality of life relative to adolescents with other psychiatric disorders [12]. Furthermore, out of all of the psychiatric disorders in adolescence, bipolar disorder carries the most significant risk of death by suicide [13].

Etiological theories of bipolar-spectrum disorders provide context for the potential links between elevated screen time and increased manic symptoms in adolescents. For example, the reward hypersensitivity model suggests that a tendency for hyperreactivity to goal-oriented and reward-related cues promotes excessive reward motivation and approach-related affectivity, leading to hypomanic/manic symptoms [14, 15]. This theory is particularly applicable to problematic screen use, which can include elements of addiction, such as mood modification, tolerance, withdrawal, and relapse [16]. Consistent with this theory, evidence suggests that screen time and problematic usage patterns in adolescents and young adults are associated with altered or heightened reward processing and less efficient cognitive control [17]. These problematic screen use patterns are also associated with shorter sleep duration and delayed bedtimes in youth, with disrupted circadian rhythms having also been suggested to underlie neurobiological vulnerability to bipolar-spectrum disorders [18, 19]. It is also plausible that greater manic symptoms could lead to excessive screen use, and bidirectional relationships exist [20]. However, despite conceptual support for a link between screen usage and manic symptoms, there have been no prospective studies of this association in adolescents.

Research in adult samples has linked bipolar disorder with patterns of smartphone use. For example, one study of university students found a significant association between bipolar disorder and addictive mobile phone use [21, 22]. Other research using objective mobile phone data found that, compared to healthy controls, individuals with bipolar disorder had greater smartphone use (daily number of text messages, duration of phone calls per day), and more severe manic symptoms were significantly associated with these smartphone variables within the bipolar disorder sample [23, 24]. Another study using objective smartphone data collected from adolescent and young adult patients with newly diagnosed bipolar disorder also found significant associations between patterns of smartphone use and manic symptoms [25]. Given the poor outcomes associated with an earlier onset of bipolar symptoms, the current study aims to offer insights into the potential prospective relationship of screen time exposure during adolescence with manic symptoms [11, 26]. Understanding this association could offer new insights in the context of adolescent and public health, including new targets for prevention/early intervention.

The current study aimed to bridge current gaps in the literature by examining the prospective relationship between screen time (Year 1) and manic symptoms (Year 3) in a diverse, nationwide sample of 10-11-year-old adolescents who participated in the Adolescent Brain Cognitive Development (ABCD) Study. Screen time was evaluated as an overall variable (i.e., total screen time) and separately as six screen subtypes: television, video games, texting, watching videos, video chatting, and social media. It was hypothesized that greater overall typical daily screen time in Year 1 would be prospectively associated with more manic symptoms in Year 3, adjusting for relevant covariates (i.e., sociodemographics, study site, data collection timeframe, attention-deficit/hyperactivity disorder (ADHD) symptoms, depressive symptoms, and manic symptoms in Year 1). There were no a priori hypotheses regarding differences in associations across the six-screen subtypes. Additionally, exploratory analyses examined problematic social media use, problematic video game use, and sleep duration (Year 2) as mediators of the prospective associations of social media screen time (Year 1) and video game screen time (Year 1) with manic symptoms (Year 3), adjusting for relevant covariates.

## Methods

This study utilized data from the Adolescent Brain Cognitive Development (ABCD) Study, a national longitudinal study of adolescent cognitive and physical health [27]. The ABCD Study applied epidemiological sampling methods to enroll 11,875 adolescents between 9 and 10 years old during the baseline period (2016–2018), aiming to represent the diversity of the adolescent population in the United States [28]. This study analyzed data from Year 1, Year 2, and Year 3 follow-ups using the ABCD 5.0 release. Out of 11,875 participants, 1,828 had missing data for the manic symptoms measure in Year 3, and 804 had missing data for covariates in Year 1, leaving a sample size of 9,243 participants for this study.

### Ethical considerations

Approval was obtained from the centralized Institutionalized Review Board (IRB) and individual study site IRB. Participant caregivers gave written, informed consent, and participants provided their written assent [28].

## Measures

### Predictor

#### Typical daily screen time (Year 1)

Screen time was obtained from the annual data of the ABCD Youth Screen Time Survey, which involves data collection and harmonizing data across years. Adolescents answered questions about typical daily time spent on six different screen use subtypes (viewing/streaming TV shows or movies, watching/streaming videos [e.g., YouTube], playing video games, texting, video chatting [e.g., Skype, FaceTime], and social media [e.g., Facebook, Instagram, Twitter]). Using validated measures [29–31], screen time was calculated separately for weekdays and weekend days. Then, we obtained typical screen time by applying a weighted average to calculate the participants’ typical weekday and weekend screen time. Typical daily screen time (overall and for each subtype) was calculated using the following formula: [(weekday average x 5) + (weekend average x 2)]/7 [32]. In the ABCD Study, self-reported screen time was significantly and positively correlated (*r* = 0.49, *p* < 0.001) with passively recorded smartphone use [33]. Likewise, prior research on self-reported social media usage showed adequate convergent validity (*r* = 0.55 to 0.65) when compared to social media measures obtained through experience sampling methods [34].

### Outcome

#### Manic symptoms (Year 3)

The ABCD study used the 7 Up subscale (7 items) of the 7 Up 7 Down Inventory to assess the presence of manic/hypomanic symptoms among individuals [35]. Adolescents responded to questions about their behaviors using a scale from (0 = Never) to (3 = very often or almost constantly). This included items such as, “Have you had periods of extreme happiness and intense energy (clearly more than your usual self) when, for several days or more, it took you over an hour to get to sleep at night?” Items were summed to create a total score. Internal consistency was good for the 7 Up subscale (α = 0.77). A clinical cutoff score of 11 or greater has previously been proposed based on its diagnostic likelihood ratio for bipolar disorder [36].

### Covariates

We included the following covariates in this study: race/ethnicity (White, Latino/Hispanic, Black, Asian, Native American, other), sex (female, male), household income [Less than $75,000 vs. more than $75,000 (approximate median household income in the U.S [37]). ], parent education (high school or less vs. college or more), participant age (years), and manic symptom score in Year 1 (α = 0.78), ADHD T-score and depressive symptoms T-score in Year 1 based on the Child Behavior Checklist DSM-Oriented Scales [38], and data collection timeframe (i.e. before vs. during the COVID-19 pandemic).

### Mediators

#### Problematic social media use and video game use (Year 2)

The Social Media Addiction Questionnaire (SMAQ) [39] and the Video Game Addiction Questionnaire (VGAQ) [39] are six-item measures that were used to assess adolescents’ self-reported problematic social media use and problematic video game use, respectively. Both questionnaires were derived from the Bergen Facebook Addiction Scale [16]. Participants who reported having at least one social media account were asked to complete the SMAQ, and those who reported video game use were asked to complete the VGAQ. On each measure, participants responded to items using a 6-point Likert-type scale ranging from 1 (Never) to 6 (Very often). The number of participants who completed the SMAQ was 4,938, while 6,736 completed the VGAQ. We assigned a value of 1 (Never) to those who reported not having a social media account or who did not play video games. A sum score was calculated for each measure, with higher scores indicating greater severity of problematic use. Internal consistency was good for the SMAQ (α = 0.89) and VGAQ (α = 0.86).

#### Munich Chronotype Questionnaire (MCTQ, Year 2)

Participants completed the MCTQ to evaluate sleep duration and behaviors, including bedtimes, sleep onset, and wake times [28]. For this analysis, we used a weighted average of sleep duration across weekdays and weekends, calculated from sleep durations reported on both free days and school days in the MCTQ.

### Statistical analyses

We assessed the associations of typical daily screen time with manic symptoms using multivariable linear regression models with robust standard error. Model 1 was not adjusted. Model 2 was adjusted for age, sex, race/ethnicity, household income, parent education, data collection site, manic symptoms in Year 1, ADHD symptoms in Year 1, depressive symptoms in Year 1, and data collection timeframe (i.e., before vs. during the COVID-19 pandemic). We also used multivariable logistic regression to assess the associations of typical daily screen time with manic symptoms (binary, based on the proposed clinical cutoff), adjusting for the same covariates.

In sensitivity analyses, we analyzed the cross-sectional associations of screen time and manic symptoms in Year 3. Prospective associations of screen time (Year 1) and clinical (binary) cutoffs of manic symptoms (Year 3) were also analyzed. The current study used generalized structural equation models with maximum likelihood estimation to test the extent to which problematic social media use mediated the association between Year 1 typical daily social media screen time and Year 3 manic symptoms. We similarly tested the extent to which problematic video game use mediated the association between Year 1 typical daily video game screen time and Year 3 manic symptoms. We also tested the extent to which sleep duration mediated the association between Year 1 total screen time and Year 3 manic symptoms. Bias-corrected (BC) 95% confidence intervals (CIs) for the indirect effect were calculated using 5000 bootstrap samples, and statistical significance was determined if the CI values did not include zero. Propensity weighting based on the American Community Survey provided by the US Census was applied to the analysis to provide representative population estimates [40]. All analyses were performed using Stata 18.0 software. Two-sided *p* < 0.05 was considered to indicate statistical significance.

## Results

Participants were 10.9 ± 0.6 years of age in Year 1; 48.8% were female, 44.0% identified as a racial or ethnic minority, 50.2% came from a household with an annual income of less than $75,000, and 89.5% had parent/caregivers with a college education or more. The mean typical daily screen time (all subtypes) was 4.6 ± 3.5 h per day (Table 1).

**Table 1:** Sociodemographic, screen time, and behavioral characteristics of 9,243 Adolescent Brain Cognitive Development (ABCD) Study participants in Year 1 (2016–2018)

Sociodemographic characteristics	Mean (SD) / %
Age (years), mean (SD)	10.9 (0.6)
Sex (%)	
Female	48.8%
Male	51.2%
Race/ethnicity (%)	
White	56.0%
Latino / Hispanic	19.1%
Black	14.4%
Asian	5.3%
Native American	3.2%
Other	2.0%
Household income (%)	
Less than $25,000	15.8%
$25,000 through $49,999	18.2%
$50,000 through $74,999	16.2%
$75,000 through $99,999	14.9%
$100,000 through $199,999	26.0%
$200,000 and greater	8.8%
Parent with a college education or more (%)	89.5%
Screen time variables, typical hours per day, mean (SD)	
Overall screen time in Year 1	4.6 (3.5)
Television shows/movies in Year 1	1.3 (1.1)
Videos (e.g. YouTube) in Year 1	1.2 (1.2)
Video games in Year 1	1.2 (1.2)
Texting in Year 1	0.4 (0.7)
Video chat in Year 1	0.3 (0.6)
Social media in Year 1	0.2 (0.6)
Overall screen time in Year 2	7.0 (5.7)
Overall screen time in Year 3	8.4 (8.2)
Manic symptoms variables	
Manic symptoms in Year 1, mean (SD)	2.3 (2.8)
Manic symptom score ≥ 11 in Year 1 (%)	1.1%
Manic symptoms in Year 3, mean (SD)	1.9 (2.5)
Manic symptom score ≥ 11 in Year 3 (%)	0.8%
Mediator variables	
Problematic social media use in Year 2, mean (SD)	6.2 (6.7) (range = 6–36)
Problematic video game use in Year 2, mean (SD)	9.1 (7.8) (range = 6–36)
Average weekly sleep duration in Year 2, hours, mean (SD)	9.2 (1.5)

Propensity weights were applied to yield representative estimates based on the American Community Survey from the US Census. SD = standard deviation

Results of adjusted models indicated that overall typical daily screen time in Year 1 was significantly associated with greater manic symptoms in Year 3 (B = 0.05, 95% CI 0.03, 0.07, *p* < 0.001). For adjusted models examining typical daily screen time for specific subtypes, videos (B = 0.14, 95% CI 0.08, 0.19, *p* < 0.001), video games (B = 0.09, 95% CI 0.04, 0.14, *p* = 0.001), texting (B = 0.18, 95% CI 0.08, 0.28, *p* < 0.001), and social media (B = 0.20, 95% CI 0.09, 0.32, *p* = 0.001) also were significantly associated with greater manic symptoms (Table 2). Only television and video chatting were found to be non-significant predictors in adjusted models.

**Table 2:** Prospective association of typical daily screen time in Year 1 and manic symptoms in Year 3 in the Adolescent Brain Cognitive Development (ABCD) Study (Youth report, *N* = 9,243)

	Model 1		Model 2	
	Unadjusted		Adjusted^a^	
	Coefficient (95% CI)	*p*	Coefficient (95% CI)	*p*
Overall screen time	**0.13 (0.11**,** 0.15)**	**< 0.001**	**0.05 (0.03**,** 0.07)**	**< 0.001**
Television shows/movies	**0.20 (0.14**,** 0.26)**	**< 0.001**	0.03 (-0.02, 0.09)	0.273
Videos (e.g., YouTube)	**0.34 (0.28**,** 0.39)**	**< 0.001**	**0.14 (0.08**,** 0.19)**	**< 0.001**
Video games	**0.23 (0.18**,** 0.28)**	**< 0.001**	**0.09 (0.04**,** 0.14)**	**0.001**
Texting	**0.45 (0.35**,** 0.55)**	**< 0.001**	**0.18 (0.08**,** 0.28)**	**< 0.001**
Video chat	**0.38 (0.27**,** 0.49)**	**< 0.001**	0.10 (-0.012, 0.21)	0.079
Social media	**0.46 (0.34**,** 0.59)**	**< 0.001**	**0.20 (0.09**,** 0.32)**	**0.001**

^a^Adjusted for age, sex, race/ethnicity, household income, parent education, study site, manic symptoms in Year 1, data collection timeframe (i.e. pre-COVID-19 or during the COVID-19 pandemic), ADHD, and depressive symptoms

In sensitivity analyses, we analyzed the cross-sectional associations of screen time and manic symptoms in Year 3 (Supplemental Table 1). We also analyzed prospective associations of screen time (Year 1) and clinical (binary) cutoffs of manic symptoms (Year 3, Supplemental Table 2).

**Fig. 1:**
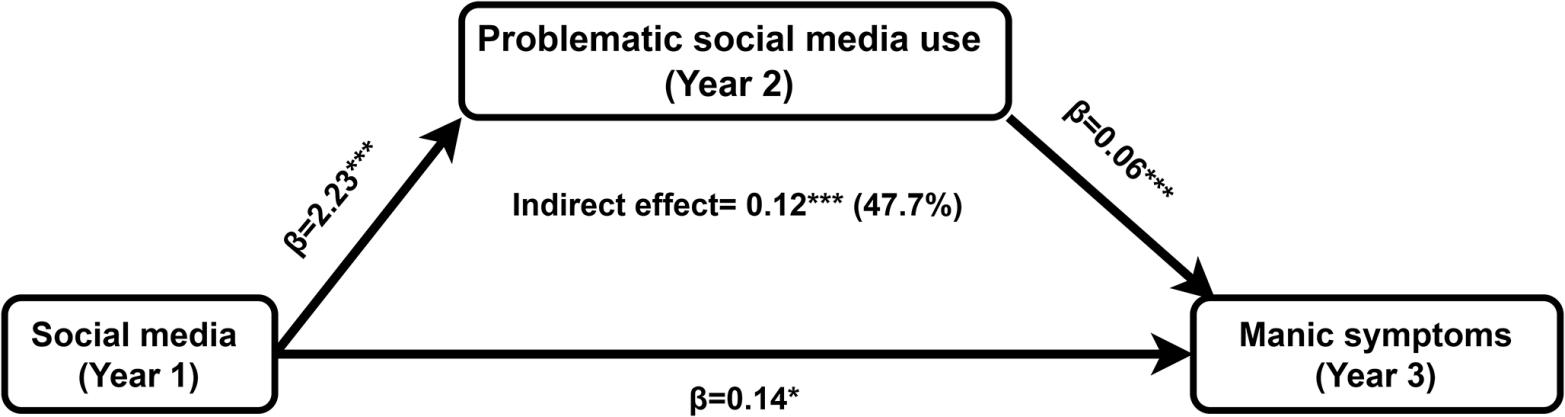
Indirect effect of social media screen time on manic symptoms through problematic social media use

Adjusting for covariates, the results of the first mediation analysis showed a significant indirect effect of typical daily social media screen time in Year 1 on manic symptoms in Year 3 through problematic social media use in Year 2 (Indirect effect, B = 0.12, 95% CI 0.09, 0.16, *p* < 0.001) (Direct effect, B = 0.14, 95%CI 0.020, 0.26, *p* = 0.026) suggesting partial mediation. (Fig. 1).

**Fig. 2:**
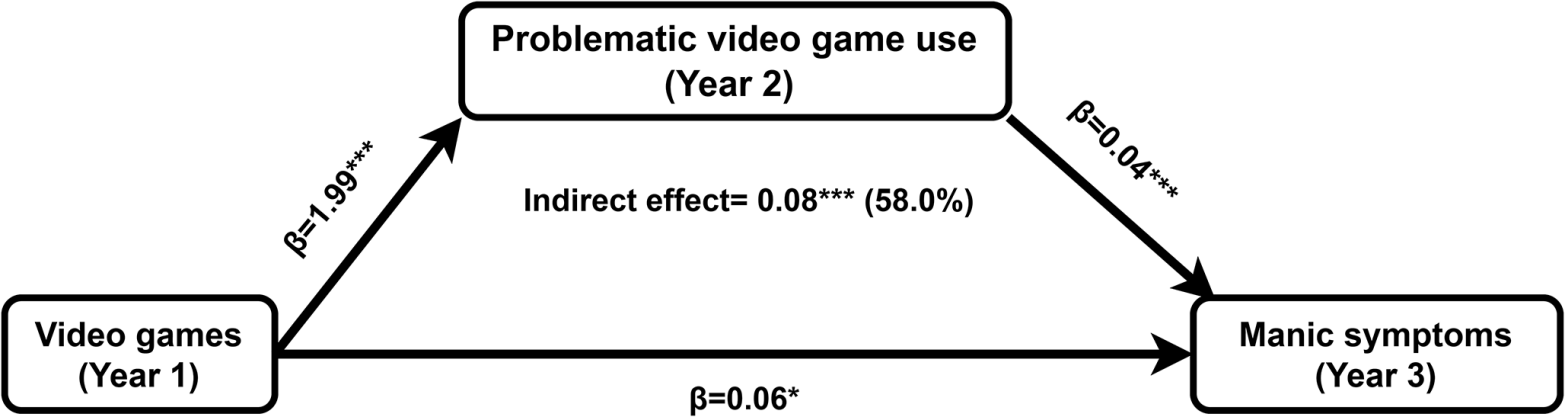
Indirect effect of video game screen time on manic symptoms through problematic video game use

Also, there was a significant indirect effect of typical daily video games screen time in Year 1 and problematic video game use in Year 2 (Indirect effect, B = 0.08, 95% CI 0.05, 0.10, *p* < 0.001), (Direct effect, B = 0.06, 95% CI 0.002, 0.11, *p* = 0.043), also suggesting partial mediation. (Fig. 2).

**Fig. 3:**
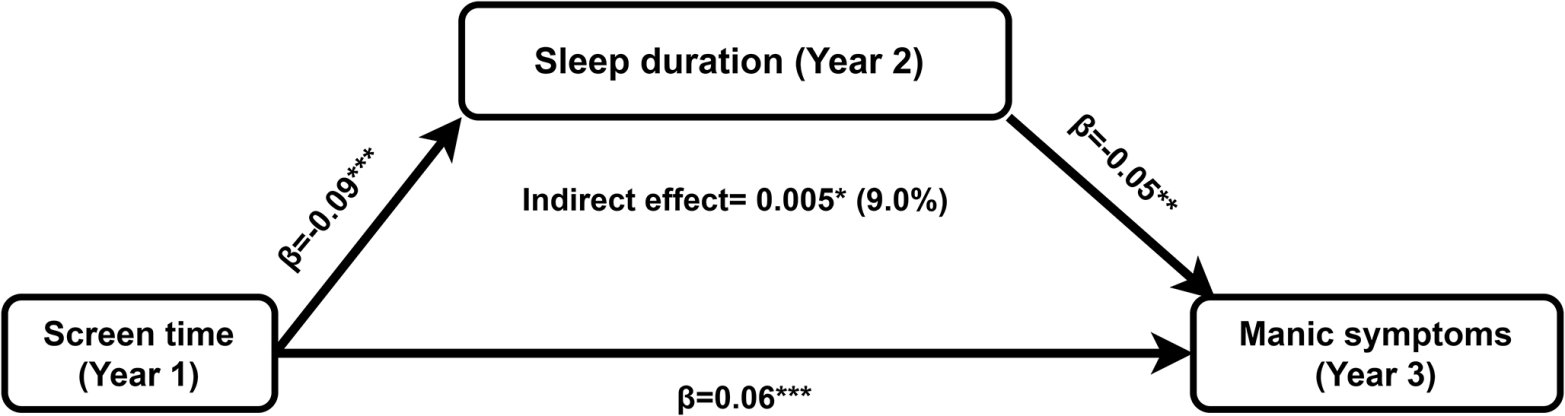
Indirect effect of total screen time on manic symptoms through sleep duration

Similarly, there was a significant indirect effect of typical daily video games screen time in Year 1 and sleep duration in Year 2 (Indirect effect, B = 0.005, 95% CI 0.001, 0.009, *p* < 0.001), (Direct effect, B = 0.06, 95% CI 0.04, 0.08, *p* < 0.001), also suggesting partial mediation (Fig. 3).

The contribution of problematic social media use, problematic video game use, and sleep duration as partial mediators of the prospective associations were 47.7% (95% CI, 23.3%, 72.0%, *p* < 0.001), 58.0% (95% CI, 31.0%, 85.0%, *p* < 0.001), and 9.0% (95% CI, 0.02%, 16.0%, *p* = 0.016), respectively (Supplementary Table 3).

## Discussion

In a demographically diverse nationwide sample of 9,243 children 10–11 years old in the United States, the current study found that greater overall typical daily screen time in Year 1 was prospectively associated with manic symptoms in Year 3, even after adjusting for confounders, including Year 1 manic symptoms. Specific screen subtypes that showed similar significant associations were videos, video games, texting, and social media, with texting and social media screen time showing the strongest prospective associations with manic symptoms when adjusting for confounders. Problematic screen use and shorter sleep duration partially mediated the prospective associations of social media screen time and video game screen time with manic symptoms.

Although this study did not examine neurobiological mechanisms of how greater screen time is prospectively associated with increased symptoms of mania, it is notable that engagement with numerous screen modalities (particularly video games and social media) provides instant gratification and feedback, stimulating the brain’s reward pathways [41, 42]. For example, video games often use reward schedules to maximize time spent playing the games, and social media platforms facilitate immediate gratification and reward through likes, comments, and other forms of social validation. Adolescents who excessively engage with social media may develop a heightened sensitivity to these rewards, leading to a cycle of compulsive use and seeking out further validation [43]. The reinforcement strategies used by most social media and video game platforms may increase dopamine release, paralleling the neurobiological processes observed in individuals experiencing manic episodes [44].

Additionally, our findings provide considerations for the role of sleep disruption in the development of mania, especially within the context of problematic screen use. Excessive gaming habits and social media use frequently coincide with irregular sleep patterns or sleep deprivation, factors known to exacerbate symptoms of mania [45]. It is plausible that the combination of heightened reward system activation and disrupted sleep and circadian rhythms may have a synergistic effect, amplifying the manifestation of manic symptoms in susceptible individuals. Adolescence is a critical developmental period marked by profound physical, emotional, and cognitive maturation. Notably, evidence suggests that adolescence is a period of vulnerability for the onset of bipolar-spectrum disorders, and earlier onset is associated with more severe and chronic outcomes compared to later onset in adulthood [46]. Understanding factors that predict the onset or worsening of manic symptoms during this developmental stage is paramount for prevention, early identification and intervention, and overall improved outcomes for affected adolescents. By elucidating the prospective associations of different forms of screen time with symptoms of mania in adolescents, our study provides valuable insights into potential risk factors that may contribute to early onset, maintenance, or exacerbation in this vulnerable age group. Moreover, given that problematic use of social media and video games partially mediated the screen time-manic symptom associations, our findings underscore the potential utility of targeted prevention/intervention programs aimed at reducing maladaptive digital engagement and fostering resilience among adolescents to mitigate the potential adverse effects of excessive screen use on mental health outcomes. One such example is the implementation of digital literacy classes in grade schools, which have demonstrated success in promoting various health-related behavior changes, with limiting screen use being among the most common behavior changes undertaken [47]. Healthcare professionals could also consider advising limits on video games, social media use, and other potentially problematic digital engagement for adolescents with mental health concerns, including manic symptoms.

Notable strengths of the current study are the large, diverse, and nationwide sample that focused on early adolescence, the prospective study design, and the test of mediation using data collected across three distinct time points. However, the study has limitations. The first was a reliance on self-report to assess screen time, which can be challenging to estimate accurately and is susceptible to potential recall biases and social desirability effects. While the social media variable specified platforms like Facebook, Instagram, and Twitter as examples, other platforms might be more pertinent to the demographic in focus. Third, although analyses adjusted for numerous potential confounding factors, other variables were not accounted for (e.g., content viewed during screen time). Additionally, reported screen time in Year 1 was not distinguished as leisure or recreational versus educational or during school hours. It is also important to note that the current study’s findings do not establish a causal effect of screen time on manic symptoms. It is also possible that greater manic symptoms could lead to greater screen time. Future research should investigate potential bidirectional relationships between screen time and manic symptoms. The majority of our participants remain below clinical thresholds for mania or hypomania and the average mania rating scale scores are low. Thus, while screen time is associated with symptoms of mania, these findings do not imply that participants are experiencing manic or hypomanic episodes. Future research could incorporate parent reports of manic and hypomanic symptoms of their children.

## Conclusions

The current study found a significant prospective association between greater typical daily screen time (overall, as well as video games, texting, social media, and video specifically) and higher symptoms of mania in a diverse national cohort of early adolescents in the United States. The effect size for overall typical daily screen time was relatively small. However, total effects are greater with more hours per day of screen exposure, and cumulative exposure to screens over several years may yield stronger associations. For social media screen time, video games screen time, and total screen time, the association with manic symptoms was partially mediated by problematic use, video game use, and sleep duration, respectively, suggesting potential pathways through which greater screen time may contribute to worsening manic symptoms. These findings highlight the importance of future research examining the behavioral, psychosocial, and neurobiological mechanisms linking excessive/problematic digital engagement and screen use to manic symptoms in adolescents, which can inform future prevention and intervention strategies.

## Electronic supplementary material

Below is the link to the electronic supplementary material.

Supplementary Material 1

## Data Availability

Data used in the preparation of this article were obtained from the ABCD Study (https://abcdstudy.org), held in the NIMH Data Archive (NDA). Investigators can apply for data access through the NDA (https://nda.nih.gov/).
