# Supplementary material for: Screen time and manic symptoms in early adolescents: prospective findings from the Adolescent Brain Cognitive Development Study
Source: Soc Psychiatry Psychiatr Epidemiol. 2025 Feb 19;60(6):1479–87. doi: 10.1007/s00127-025-02814-6 (PMC12162243; doi:10.1007/s00127-025-02814-6)
Supplement: Supplementary file 1 — Supplementary Material 1 [file 127_2025_2814_MOESM1_ESM.docx]

**Supplementary Information**

**Article:** Screen Time and Manic Symptoms in Early Adolescents: Prospective Findings from the Adolescent Brain Cognitive Development Study

**Journal:** Social Psychiatry and Psychiatric Epidemiology

**Authors and affliations:** Jason M. Nagata, M.D., M.Sc.,^1*^ Gabriel Zamora,^1*^ Abubakr A.A. Al-Shoaibi, Ph.D.,^1^ Jason M. Lavender, Ph.D.,^2,3^ Kyle T. Ganson, Ph.D., M.S.W.,^4^ Alexander Testa, Ph.D.,^4^ Jinbo He, Ph.D.,^6^ Fiona C. Baker, Ph.D.^7,8^

*Authors contributed equally

^1^ Department of Pediatrics, University of California, San Francisco, San Francisco, California, USA

^2^ Military Cardiovascular Outcomes Research Program (MiCOR), Department of Medicine, Uniformed Services University of the Health Sciences, Bethesda, MD, USA

^3^ The Metis Foundation, San Antonio, TX, USA

^4^ Factor-Inwentash Faculty of Social Work, University of Toronto, Toronto, Ontario, Canada

^5^ Department of Management, Policy and Community Health, University of Texas Health Science Center at Houston, Houston, Texas, USA

^6^ Division of Applied Psychology, School of Humanities and Social Science, The Chinese University of Hong Kong, Shenzhen, Guangdong, China

^7^ Center for Health Sciences, SRI International, Menlo Park, California, USA

^8^ School of Physiology, University of the Witwatersrand, Johannesburg, South Africa

**Corresponding Author:**

Jason M. Nagata, M.D., M.Sc.,

University of California, San Francisco

E-mail: [jason.nagata@ucsf.edu](mailto:jason.nagata@ucsf.edu)

Supplemental Table 1. Cross-sectional (Year 3) associations of typical daily screen time and manic symptoms in the Adolescent Brain Cognitive Development (ABCD) Study (Youth report, N=9,014)

|  | Model 1 | | Model 2 |  |
| --- | --- | --- | --- | --- |
|  | Unadjusted | | Adjusted |  |
|  | Coefficient (95% CI) | p | Coefficient (95% CI) | p |
| Overall screen time | **0.07 (0.06, 0.08)** | **<0.001** | **0.04 (0.03, 0.05)** | **<0.001** |
| Television shows/movies | **0.17 (0.14, 0.21)** | **<0.001** | **0.07 (0.04, 0.11)** | **<0.001** |
| Videos (e.g., YouTube)* | **-** | **-** | **-** | **-** |
| Video games | **0.12 (0.09, 0.14)** | **<0.001** | **0.06 (0.04, 0.08)** | **<0.001** |
| Texting | **0.21 (0.16, 0.25)** | **<0.001** | **0.18 (0.16, 0.25)** | **<0.001** |
| Video chat | **0.22 (0.16, 0.27)** | **<0.001** | **0.13 (0.08, 0.18)** | **<0.001** |
| Social media | **0.19 (0.15, 0.23)** | **<0.001** | **0.11 (0.07, 0.14)** | **<0.001** |

Model 1: unadjusted

Model 2: Adjusted for age, sex, race/ethnicity, household income, parent education, study site, manic symptoms at Year 1, COVID-19 pandemic timeframe (before vs during) at year 3, ADHD, and depressive symptoms

*Videos time was not assessed in Year 3.

| Supplemental Table 2. Prospective association of typical daily screen time at Year 1 and manic symptoms (binary clinical cutoff) at Year 3 in the Adolescent Brain Cognitive Development (ABCD) Study (Youth report, N=9,227) | | | | |
| --- | --- | --- | --- | --- |
|  | Model 1 | | Model 2 | |
|  | Unadjusted | | Adjusted^a^ | |
|  | OR (95% CI) | p | OR (95% CI) | p |
| Overall screen time | **1.11 (1.07, 1.16)** | **<0.001** | **1.06 (1.00, 1.11)** | **0.032** |
| Television shows/movies | 1.09 (0.90, 1.30) | 0.391 | 0.93 (0.76, 1.12) | 0.436 |
| Videos (e.g., YouTube) | **1.37 (1.17, 1.61)** | **<0.001** | 1.15 (0.96, 1.37) | 0.126 |
| Video games | **1.29 (1.11, 1.50)** | **0.001** | 1.12 (0.93, 1.34) | 0.242 |
| Texting | **1.45 (1.20, 1.76)** | **<0.001** | 1.22 (0.99, 1.48) | 0.051 |
| Video chat | **1.46 (1.20, 1.78)** | **<0.001** | 1.21 (0.97, 1.51) | 0.086 |
| Social media | **1.55 (1.27, 1.88)** | **<0.001** | **1.35 (1.09, 1.67)** | **0.005** |
| ^a^ Adjusted for age, sex, race/ethnicity, household income, parent education, study site, manic symptoms at Year 1, COVID-19 pandemic timeframe (before vs during) at year 3, ADHD, and depressive symptoms | | | | |

Supplementary Table 3. Direct and indirect effects of total screen time, social media, and video games (predictor variables) on manic symptoms (outcome variable) through problematic social media use, problematic video games use, and sleep duration (mediator variables)

|  | Direct effect | | Indirect effect | | Mediated proportion | |
| --- | --- | --- | --- | --- | --- | --- |
| Predictor Variables | B (95% CI) | p | B (95% CI) | p | % (95%CI) | p |
| Social media screen time (Year 1) | **0.14 (0.02, 0.26)** | **0.026** | **0.12 (0.09, 0.16)** | **<0.001** | **47.7 (23.3, 72.0)** | **<0.001** |
| Video games screen time (Year 1) | **0.06 (0.002, 0.11)** | **0.043** | **0.08 (0.05, 0.10)** | **<0.001** | **58.0 (31.0, 85.0)** | **<0.001** |
| Total screen time (Year 1) | **0.06 (0.04, 0.08)** | **<0.001** | **0.005 (0.001, 0.009)** | **0.010** | **9.0 (0.02, 0.16)** | **0.016** |

Adjusted for age, sex, race/ethnicity, household income, parent education, study site, manic symptoms at Year 1, COVID-19 pandemic timeframe (before vs during) at year 3, ADHD, and depressive symptoms
